# Understanding the intracellular trafficking and intercellular transport of potexviruses in their host plants

**DOI:** 10.3389/fpls.2014.00060

**Published:** 2014-03-18

**Authors:** Mi-Ri Park, Rae-Dong Jeong, Kook-Hyung Kim

**Affiliations:** ^1^Department of Agricultural Biotechnology, Seoul National UniversitySeoul, South Korea; ^2^Plant Genomics and Breeding Institute, Seoul National UniversitySeoul, South Korea; ^3^Research Institute for Agriculture and Life Sciences, Seoul National UniversitySeoul, South Korea; ^4^Advanced Radiation Technology Institute, Korea Atomic Energy Research InstituteJeongeup, South Korea

**Keywords:** potexvirus, intracellular trafficking, cell-to-cell movement, movement complexes, plasmodesmata, host proteins, host cellular membranes

## Abstract

The movement of potexviruses through the cytoplasm to plasmodesmata (PD) and through PD to adjacent cells depends on the viral and host cellular proteins. Potexviruses encode three movement proteins [referred to as the triple gene block (TGB1–3)]. TGB1 protein moves cell-to-cell through PD and requires TGB2 and TGB3, which are endoplasmic reticulum (ER)-located proteins. TGB3 protein directs the movement of the ER-derived vesicles induced by TGB2 protein from the perinuclear ER to the cortical ER. TGB2 protein physically interacts with TGB3 protein in a membrane-associated form and also interacts with either coat protein (CP) or TGB1 protein at the ER network. Recent studies indicate that potexvirus movement involves the interaction between TGB proteins and CP with host proteins including membrane rafts. A group of host cellular membrane raft proteins, remorins, can serve as a counteracting membrane platform for viral ribonucleoprotein (RNP) docking and can thereby inhibit viral movement. The CP, which is a component of the RNP movement complex, is also critical for viral cell-to-cell movement through the PD. Interactions between TGB1 protein and/or the CP subunit with the 5′-terminus of genomic RNA [viral RNA (vRNA)] form RNP movement complexes and direct the movement of vRNAs through the PD. Recent studies show that tobacco proteins such as NbMPB2C or NbDnaJ-like proteins interact with the stem-loop 1 RNA located at the 5′-terminus of *Potato virus X* vRNA and regulate intracellular as well as intercellular movement. Although several host proteins that interact with vRNAs or viral proteins and that are crucial for vRNA transport have been screened and characterized, additional host proteins and details of viral movement remain to be characterized. In this review, we describe recent progress in understanding potexvirus movement within and between cells and how such movement is affected by interactions between vRNA/proteins and host proteins.

## INTRODUCTION

The infection cycle of plant RNA viruses includes the invasion of the host plant, RNA replication, cell-to-cell and long-distance movement in the host, and release from the host. Because their genomes are small and encode only a few genes, plant RNA viruses utilize many host factors during the infection cycle. The replication of viral RNAs (vRNAs) in host plants has been frequently studied but virus movement and other aspects of the viral infection cycle have received less attention (Mackenzie, 2005; Nagy and Pogany, 2010).

**FIGURE 1:**
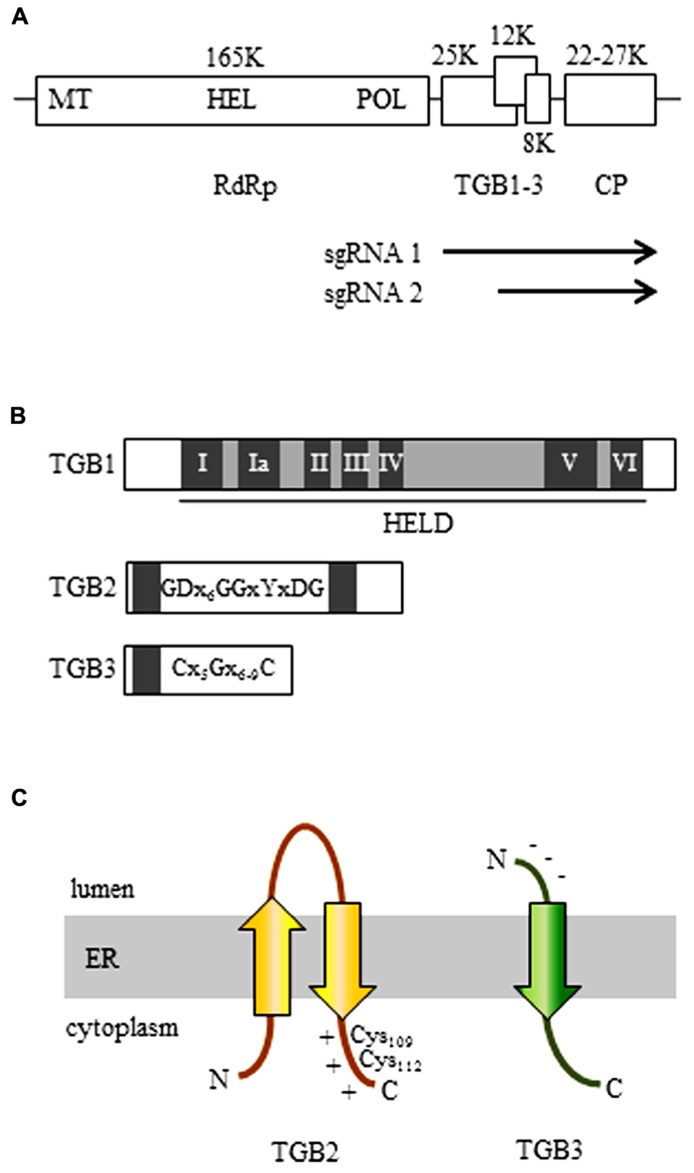
**The organization of the potexvirus genome. (A)** The RNA-dependent RNA polymerase (RdRp, replicase) gene contains a methyltransferase domain (MET), a helicase domain (HEL), and an RNA polymerase domain (POL). The three genes of the triple gene block (TGB) are partially overlapped. Arrows indicate subgenomic (sg) RNAs for expression of TGBs. **(B)** The organization of the three TGB genes. TGB1: The first TGB ORF encodes the TGB1 protein and has a helicase-like domain (HELD), which contains seven typical motifs of a general helicase (I, Ia, II, III, IV, V, and VI; dark boxes). TGB2: the TGB2 protein is encoded in the second TGB ORF and has two transmembrane domains (dark boxes). The GDx_6_GGxYxDG sequence is conserved in TGB2-encoding viruses. TGB3: The TGB3 protein is encoded by the third TGB ORF and contains a transmembrane domain (dark box). Among the TGB3-encoding potexviruses, the TGB3 gene has a conserved C(x_5_)G(x_6__-__9_)C sequence. **(C)** 3-D structural models of TGB2 and TGB3 protein in the endoplasmic reticulum (ER). Potexvirus TGB2 protein integrates into ER membranes to form a U-like structure (with the central loop exposed to the ER lumen and with both the N- and C-terminus located in the ER cytoplasm) and has two conserved cysteine residues (Cys_109_ and Cys_112_) in the C-terminal region, which has a positive net charge (TGB2). Potexvirus TGB3 has an N-terminus that has a negative net charge and is exposed to the ER lumen and a C-terminus that is exposed to the ER cytoplasm (Morozov and Solovyev, 2003).

Potexviruses have been extensively studied and belong to the *Alphaflexiviridae*, a new family of plant RNA viruses, among which the genomes of the genus *Potexvirus* contain five open reading frames (ORFs) encoding an RNA-dependent RNA polymerase (RdRp; replicase), three overlapping movement proteins (MPs) [called the triple gene block (TGB1–3)], and the coat protein (CP; Adams et al., 2004; King et al., 2009; **Figure 1A**). All five of the virus-encoded proteins are used either in viral replication or in movement in infected host plants (Verchot-Lubicz, 2005; Verchot-Lubicz et al., 2010; Solovyev et al., 2012; Park et al., 2013). At the initial stage of infection, potexviruses, which have a plus (+)-stranded RNA genome, release vRNA from the virion and produce the virus-encoded replicase using host translation machinery. Replicase then forms a viral replication complex (VRC) along with several host factors and subsequently synthesizes (i) minus (-)-stranded vRNA from (+) vRNA and (ii) (+) vRNA or (+) subgenomic (sg) RNA from synthesized (-) vRNA. CP and TGB1–3 proteins are translated from the synthesized (+) sgRNAs and are used for encapsidation and movement of their progeny (+) vRNAs, which were produced from (-) vRNA as template, into neighboring uninfected cells through the plasmodesmata (PD). The movement of plant viruses is by definition essential if the progeny (+) vRNAs or virions are to spread into neighboring uninfected cells. In moving the progeny (+) vRNAs or virions via PD into adjacent cells, most plant viruses use their own MP(s). For potexviruses, substantial research has determined that viral cell-to-cell movement requires TGB proteins and CP (Chapman et al., 1992; Forster et al., 1992; Baulcombe et al., 1995; Morozov and Solovyev, 2003; Verchot-Lubicz et al., 2010; Niehl and Heinlein, 2011; Schoelz et al., 2011; Solovyev et al., 2012).

Most TGB-encoding plant RNA viruses belong to either the *Virgaviridae* or the *Flexiviridae *of the *Alphaflexiviridae* and *Betaflexiviridae* (Verchot-Lubicz et al., 2010). Recently, Solovyev et al. (2012) summarized information concerning TGB proteins and TGB-mediated plant viruses. The TGB proteins have been classified into two major groups, i.e., potex- and hordei-like TGBs, based on phylogeny and on differences in the viral movement mechanism (Morozov and Solovyev, 2003; Verchot-Lubicz et al., 2010). The potex-like viruses form filamentous virions containing a monopartite RNA genome and require the CP for cell-to-cell movement, whereas hordei-like viruses are rod-shaped, have multipartite RNA genomes, and do not require the CP for cell-to-cell movement (Morozov and Solovyev, 2003; Adams et al., 2004; Martelli et al., 2007; Verchot-Lubicz et al., 2010). Verchot-Lubicz et al. (2010) summarized and compared the movement strategies employed by TGB proteins in potex-like viruses and hordei-like viruses. In the current review, we supplement these earlier reviews by considering more recent findings on cell-to-cell movement of potexvirus vRNA and/or virions through the PD including the intracellular trafficking and intercellular transport of vRNA. We also describe the unique function of potexvirus RNA elements during intracellular trafficking.

## THREE TGB PROTEINS (TGB1, TGB2, AND TGB3)

First, we summarize the general information concerning potexvirus TGB proteins, which are required for vRNA trafficking through PD in host cells. Most potex-like viruses belonging to the genera *Potexvirus*, *Allexivirus*, and *Mandarivirus* of the family *Alphaflexiviridae* and the genera *Carlavirus* and *Foveavirus *of the family *Betaflexiviridae* encode three TGB proteins (Adams et al., 2004; King et al., 2009). The other TGB protein-containing viruses belong to the genera *Hordeivirus*, *Pomovirus*, *Pecluvirus*, and *Benyvirus *(Morozov and Solovyev, 2003; Adams et al., 2004). Among these nine genera, three partially overlapping ORFs encode TGB1–3 proteins and are usually expressed from two sgRNAs (Zhou and Jackson, 1996; Verchot et al., 1998; **Figure 1A**). In the TGB-containing potexviruses, TGB1 protein is translated from sgRNA1, whereas TGB2 and TGB3 proteins are co-translated from sgRNA2 (Verchot et al., 1998). *Barley stripe mosaic virus *(BSMV), in the genus *Hordeivirus*, temporally controls the level of TGB proteins accumulation so that the estimated ratio is 100:10:1 (TGB1:TGB2:TGB3) during replication in plant cells (Johnson et al., 2003; Jackson et al., 2009). Although the ratio of TGB proteins for potexviruses has not been reported, according to the evidence from BSMV, it seems that TGB-encoding plant viruses control the expression level of TGB proteins by controlling the synthesis of sgRNAs.

Potexvirus TGB1 protein is encoded by the first TGB ORF and contains a helicase-like domain (HELD) that has seven conserved typical motifs (I, Ia, II, III, IV, V, and VI) in superfamily 1 (SF1) among three SFs (SF1, SF2, and SF3) of RNA helicases (Gorbalenya and Koonin, 1993; Liou et al., 2000; Kalinina et al., 2002; Lin et al., 2004; Leshchiner et al., 2006; Han et al., 2007). Motif I has conserved GKS/T tripeptides in potexviruses, and motif II is responsible for binding ATP and Mg^2^^+^ to corresponding sites (“Walker A” and “Walker B”) found in numerous ATP-binding proteins (Gorbalenya and Koonin, 1993; Kadare and Haenni, 1997). Potexvirus TGB1 protein also functions as a suppressor of RNA silencing (Wung et al., 1999; Voinnet et al., 2000; Bayne et al., 2005; Lim et al., 2010b) and as a translational activator (Atabekov et al., 2000; **Figure 1B**; TGB1). Especially, a previous study suggested that TGB1 of *Bamboo mosaic virus* (BaMV) has RNA-binding activity, which might be associated with RNA silencing (Wung et al., 1999). In *Potato virus X* (PVX), it was shown that TGB1 mutants that lack ability to suppress RNA silencing are not able to contribute to viral movement, whereas some PVX TGB1 mutants that are movement defective still function as RNA silencing suppressor, indicating that these mechanisms of potexvirus TGB1 are integrated (Bayne et al., 2005). Interestingly, Senshu et al. (2009) showed different levels of the RNA silencing suppression by TGB1 in potexviruses including PVX, *Plantago asiatica mosaic virus*, *Asparagus virus 3,* *White clover mosaic virus* (WClMV), and *Tulip virus X*. These results indicate that potexvirus TGB1s contribute various levels in suppressing RNA silencing. In contrast, another study has shown that a single mutation in TGB1 from *Alternanthera mosaic virus* (AltMV) that showed a dramatic reduction of RNA silencing suppression activity still supports full cell-to-cell movement, indicating that potexvirus TGB1 protein appears to be uncoupled in silencing suppression and movement functions (Lim et al., 2010c). By using green fluorescent protein (GFP) fusion TGB1 of PVX, researchers have shown that, in addition to having RNA-binding and helicase activities, TGB1 protein increases the PD size-exclusion limits (SELs) for viral cell-to-cell movement (Angell et al., 1996; Tamai and Meshi, 2001).

Triple gene block 1-mediated X-body reorganization contributes to the compartmentalization of the viral gene products during viral infection (Tilsner et al., 2012; Yan et al., 2012). A TGB1 mutant that lost the movement function could not form rod-like structures, whereas those mutants that still supported cell-to-cell movement formed rod-like structures, indicating that TGB1’s movement function is closely associated with its function in the formation of rod-like structures (Yan et al., 2012). In addition, Tilsner et al. (2012) showed that the PVX TGB1 protein reorganizes actin and endomembranes (the endoplasmic reticulum [ER] and golgi) into the X-body as a VRC.

Potexvirus TGB2 protein is encoded by the second ORF of the TGB gene cluster and is an integral membrane protein that has two predicted transmembrane domains that interact with ER membranes (Mitra et al., 2003; Morozov and Solovyev, 2003). A topological study with BaMV showed that potexvirus TGB2 protein integrates into the ER membranes in a U-like structure with the central loop exposed to the ER lumen and with both the N- and C-terminus located on the cytoplasmic side of the ER (Hsu et al., 2008; **Figure 1C**; TGB2). Cysteine-to-alanine substitution analysis indicated that two conserved cysteine residues (Cys_109_ and Cys_112_) in the C-terminal region of potexvirus TGB2 protein are critical for both cell-to-cell and systemic movement of BaMV (Hsu et al., 2008; Tseng et al., 2009). In addition, potexvirus TGB2 protein has sequence-independent RNA-binding activity (Cowan et al., 2002; Hsu et al., 2009).

Like TGB2, potexvirus TGB3 protein, which is encoded by the third TGB ORF, is also an integral protein in ER membranes (Krishnamurthy et al., 2003; **Figure 1C**; TGB3). TGB3 protein has a conserved C(x_5_)G(x_6__-__9_)C sequence and a predicted transmembrane domain (**Figure 1B**; TGB3). The N-terminus of the TGB3 protein has a negative net charge and is exposed to the ER lumen, while the C-terminus is exposed to the cytoplasmic side of the ER (Krishnamurthy et al., 2003; Morozov and Solovyev, 2003; **Figure 1C**; TGB3). The cytoplasmic tail (C-terminus) of TGB3 protein contains a sorting signal that is necessary for TGB3 oligomerization and for the targeting of integral membrane proteins to cortical ER tubules (Wu et al., 2011). The TGB3 sorting signal is highly conserved among potexviruses, and a TGB3 mutant defective in the sorting signal showed impaired cell-to-cell viral movement (Wu et al., 2011).

Mutational analyses have shown that localization of TGB2 and TGB3 proteins into the ER is critical for viral cell-to-cell movement (Krishnamurthy et al., 2003; Mitra et al., 2003). Both TGB2 and TGB3 proteins enhanced cell-to-cell diffusion of free GFP or GFP–sporamin fusion proteins through the PD (Tamai and Meshi, 2001; Haupt et al., 2005), suggesting that TGB2 and TGB3 proteins are capable of gating the PD.

## INTRACELLULAR TRAFFICKING OF POTEXVIRUS vRNA FROM THE CYTOPLASM TO PD

**FIGURE 2:**
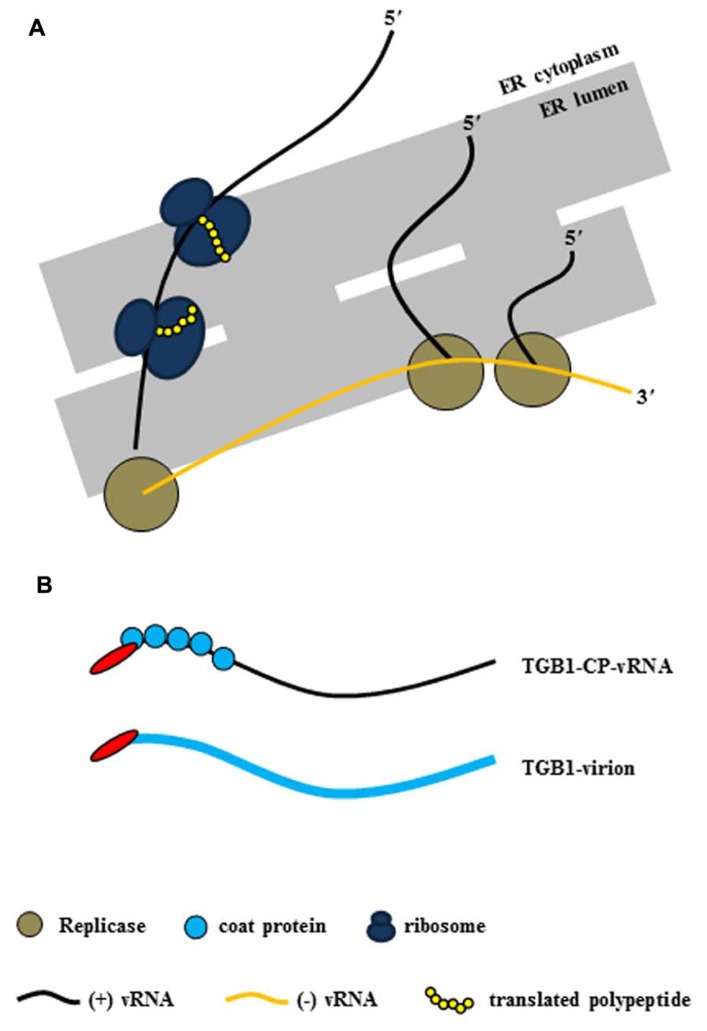
**Virus replication complex (VRC) and RNP movement complex in the ER tubule. (A)** Potexvirus constructs the VRC, which consists of vRNA, replicase, and host proteins (i.e., ribosomes) for replication of vRNA. Replicase (brown circles) produces progeny (+) vRNA from (-) vRNA as a template in the ER structure, and host ribosomes (dark blue) translate viral proteins (yellow chain) from progeny (+) vRNA. **(B)** The two kinds of RNP movement complexes in potexviruses. In one case, the PD-transportable form of potexvirus vRNA is partially encapsidated by the CP subunit, and TGB1 protein is associated with the 5′ end of the partially CP-coated vRNA (TGB1-CP-vRNA). In the second case, TGB1 protein binds to the CP-encapsidated virion (TGB1-virion). The blue circles indicate CP in the first case, and the thick blue line indicates CP and virion in the second case.

As mentioned earlier, potexvirus TGB1 protein has RNA-binding and helicase activities (Kalinina et al., 2002). Potexviruses form VRC containing (-) vRNA as templates, viral replicase, and host protein(s) at the host cellular membranes (**Figure 2A**). Following the replication of (+) vRNA by replicase, (+) vRNA is converted to the PD-transportable potexvirus vRNA form by TGB1 protein for the cell-to-cell movement through the PD. Two models have been proposed for the formation of PD-transportable potexvirus vRNA (intact virion or a ribonucleoprotein [RNP] movement complex containing vRNA, TGB1 protein, and CP) during the cell-to-cell movement of vRNA through the PD. Lough et al. (2000) provided experimental evidence that vRNAs of potexviruses including PVX and WClMV were transported by the formation of RNP movement complex involving vRNA, TGB1 protein, and CP rather than intact virion alone. Recently, however, Verchot-Lubicz et al. (2010) and Tilsner et al. (2013) have provided evidence that the PD-transportable potexvirus vRNA form is partially or fully encapsidated by the CP subunit and that the TGB1 protein is associated with the 5′ end of the CP-coated vRNA. Therefore, the formation of the RNP movement complex involves the association of the TGB1 protein with the 5′ end of the CP-coated vRNA (Verchot-Lubicz et al., 2010; Tilsner et al., 2013; **Figure 2B**).

**FIGURE 3:**
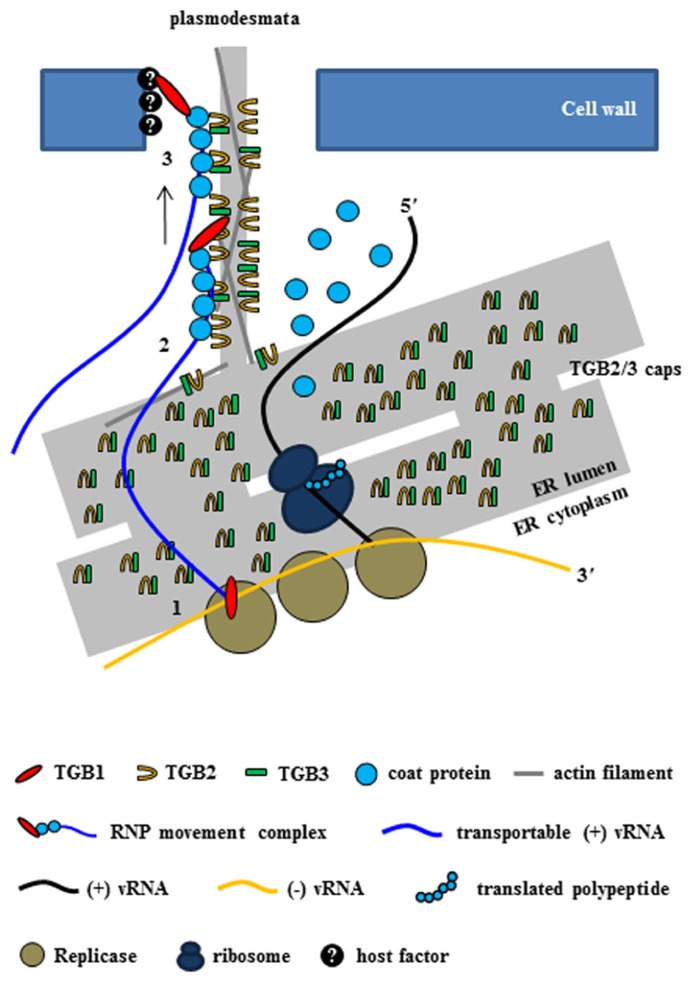
**Intercellular transport of potexvirus vRNA to PD at the late stage of viral infection.** The PD-transportable potexvirus vRNA (blue line) is released from the intermediate double-stranded RNA form by TGB1 protein acting as an RNA helicase (1). The PD-transportable potexvirus vRNA is partially or fully encapsidated by CP subunits, and then TGB1 protein associates with the 5′ end of the partially or fully CP-coated vRNA for the formation of the RNP movement complex at PD “cap” structures that consist of TGB2, TGB3 proteins, and VRC at the ER adjacent to the PD orifice (2). Then, the TGB1-driven RNP movement complex directs the nascent virions into the PD pore (3, Tilsner et al., 2013).

As mentioned earlier, cell-to-cell movement of potexvirus vRNA through the PD requires the three TGB proteins and the CP. Several host proteins might also be required for the formation of the RNP movement complex, but how host proteins cooperate with the RNP movement complex is unclear. The RNP movement complex containing TGB1 protein, CP, and vRNA subsequently binds to other viral proteins (TGB2 and TGB3 proteins) and possibly with several host proteins in the ER tubule (**Figure 3**).

Potexvirus TGB1 protein is a component in the RNP movement complex (TGB1-CP-vRNA or TGB1-virion) and is able to transport vRNA into the PD as well as provide RNA helicase activity probably near the PD. However, plant expression experiments using GFP–PVX TGB1 to determine the movement of TGB1 protein within and between cells indicated that GFP–TGB1 was diffused throughout the cytoplasm, nucleoplasm, and rod-shape structure (Howard et al., 2004; Samuels et al., 2007). These findings indicate that potexvirus TGB1 protein requires viral CP in the RNP movement complex to move together with their vRNA into PD.

In addition, Rodionova et al. (2003) have shown that PVX TGB1 protein, which is translated from sgRNA at an early stage of viral infection, binds to polar CP subunits located at the end of PVX virion, resulting in the disassembling of the CP particles of the virion. This creates a translatable form of PVX vRNA in host plants. It seems that potexvirus TGB1 protein directly binds to CP but not to vRNA for disassembly of the PVX virion and thereby enables the release of a translatable form of vRNA. In the case of *Plantago asiatica mosaic virus*, the TGB1 protein directly interacts with CP and binds with the 5′ non-translated region (NTR) of vRNA for virion formation or for formation of the RNP movement complex (Ozeki et al., 2009).

**FIGURE 4:**
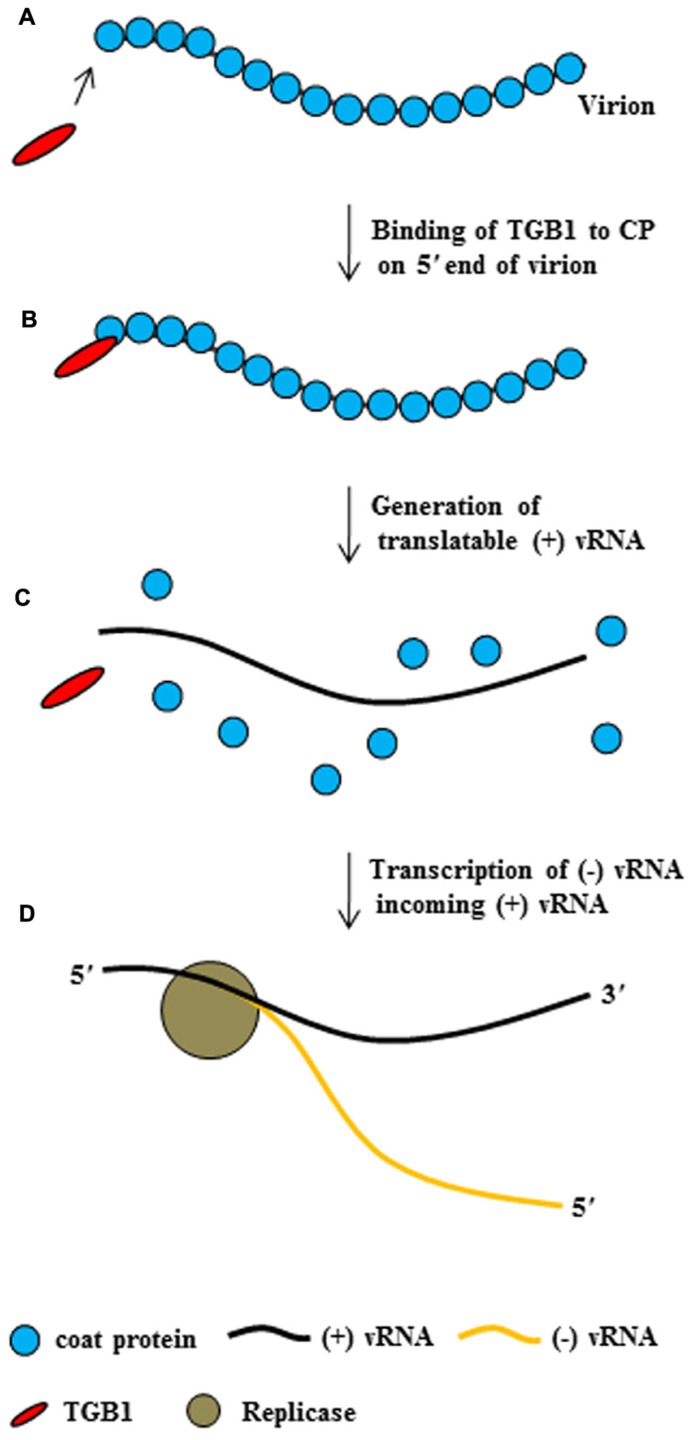
**The conversion of potexvirus virions into translatable vRNA.** PVX TGB1 protein binds to polar CP subunits located at the end of PVX virion **(A,B)**. Then, binding of TGB1 protein causes the CP to separate from the PVX virion **(C)** early in infection after movement of virion into uninfected adjacent cells, such that the virion become translatable. Replicase then replicates (-) vRNA from the translatable form of PVX (+) vRNA **(D)**, replicase transcribed (-) vRNA from (+) vRNA, and finally (not shown), ribosomes bind to the (+) vRNA.

**FIGURE 5:**
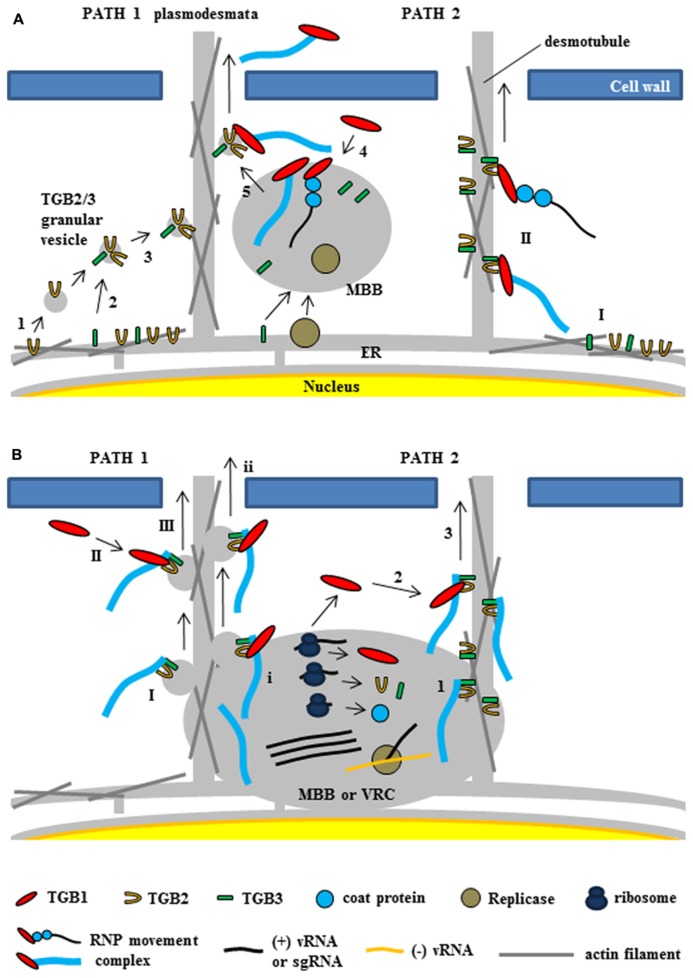
**Two models for intracellular trafficking of potexviruses.** These two models were partially adapted from Chou et al. (2013) and Verchot-Lubicz et al. (2010). **(A)** The first model proposed by Verchot-Lubicz et al. (2010). (PATH 1) Potexvirus RNP movement complex is trafficked by TGB2-induced granular vesicles with TGB3 protein (TGB2/3 granular vesicles) to the PD. TGB2 protein is able to induce the formation of granular vesicles derived from the ER membranes (1) and colocalizes with TGB3 protein (2). TGB3 protein directs movement of the TGB2-induced granular vesicles from the perinuclear ER to the cortical ER (3). vRNA or virion form of potexvirus mainly associates with membrane-bound bodies (MBB) derived the perinuclear ER that contains replicase and TGB3 protein. TGB1-bound vRNA or virion form (RNP movement complex released from MBB by TGB3 protein (4) and binds to TGB2/3 granular vesicles in the ER tubule for trafficking vRNA through the PD (5). (PATH 2) TGB2 protein localizes in the ER tubules with TGB3 protein, which can alone localize in membrane bodies at the cell periphery (I), and then RNP movement complex binds to TGB2 and TGB3 protein for vRNA trafficking to the PD (II). **(B)** The second model for TGB3-based intracellular trafficking of vRNA in BaMV (Chou et al., 2013). (PATH 1) Virion is associated by TGB2/3 granular vesicles (I) from ER-derived MBB or viral replication complex (VRC). Cytosolic TGB1 (II) or TGB1 protein (i) interacts with TGB2 protein into virion-TGB2/3 granular vesicles in cytosol and MBB or VCR, respectively and leads vRNA toward the PD (III and ii). (PATH 2) Virion interacts with TGB3 protein of TGB2/3 complex (1) and cytosolic TGB1 protein (2) interacts with TGB2 protein in virion-TGB2/3 complex. TBG1 protein drives virion-TGB1/2/3 complex toward the PD (3).

Taken together, these findings suggest that potexvirus TGB1 protein may have at least two functions during the virus infection cycle. First, TGB1 protein might convert non-translatable vRNA into a translatable form. To accomplish this first function, TBG1 protein early in infection in uninfected adjacent cells binds to the end of CP subunits on the virion (**Figures 4A,B**), leading to the disassembling of the CP from vRNA and the releasing of a translatable form of vRNA (**Figure 4C**). Finally, replicase transcribes (-) vRNA for incoming (+) RNA (**Figure 4D**). Second, TGB1 protein might facilitate the transport of vRNA to the PD. To accomplish this second function, TGB1 protein binds to the transportable form (virion form or partial CP-encapsidated vRNA) of progeny vRNA, which is generated after vRNA accumulates early in infection, and along with other viral proteins becomes part of the RNP movement complex (**Figures 3** and **5**).

Potexvirus CP, which is another component in the RNP movement complex, is required for viral movement as well as for genome encapsidation in plants. Potexvirus CP mutants that were defective for interaction with TGB1 protein were able to form virus particles *in vitro* but were unable to move from cell-to-cell (Zayakina et al., 2008; Ozeki et al., 2009; Tilsner et al., 2012). This result suggests that the interaction between TGB1 protein and CP mediates the movement of the potexvirus RNP movement complex to the PD.

As mentioned earlier, research with GFP–TGB2 and GFP–TGB3 fusion proteins has demonstrated that both TGB2 and TGB3 proteins are integral membrane proteins in the ER or ER-associated vesicles located at actin filaments (Morozov and Solovyev, 2003; Hsu et al., 2008). In relation to the role of TGB2 and TGB3 proteins for potexvirus vRNA trafficking to PD, two models have been proposed (Verchot-Lubicz et al., 2010; Chou et al., 2013). Verchot-Lubicz et al. (2010) summarized the first model suggesting two pathways of potexvirus vRNA trafficking to PD based on the interactions between TGB2 and TGB3 proteins (**Figure 5A**, PATH 1 and PATH 2). One pathway suggests that the potexvirus RNP movement complex is transported by TGB2-induced granular vesicles as directed by TGB3 protein (TGB2/3 granular vesicles) to PD (**Figure 5A**, PATH 1). In support of this pathway, TGB2 protein is able to induce the formation of granular vesicles derived from the ER membranes that associate with actin filaments (Ju et al., 2005). Mutational analyses have shown that these TGB2-induced granular vesicles are necessary for cell-to-cell movement of PVX (Ju et al., 2007). Although subcellular localization assays have shown that PVX TGB3 protein is localized to the ER tubules when expressed alone in tobacco plant cells (Ju et al., 2005), other studies have shown that TGB3 protein is co-localized at the TGB2-induced granular vesicles in PVX-infected plant cells, suggesting that TGB2 protein is responsible for the localization of TGB3 protein at the ER membrane-associated granular vesicles (Schepetilnikov et al., 2005). TGB3 protein directs the movement of the TGB2-induced granular vesicles from the perinuclear ER to the cortical ER in both yeast and plant systems (Lee et al., 2010; Wu et al., 2011). These results indicate that potexvirus TGB3 protein serves as a driving factor for movement of vRNA to PD via TGB2-induced granular vesicles (**Figure 5A**, PATH 1).

PVX vRNA mainly associates with membrane-bound bodies (MBB) from the perinuclear ER that contain replicase and TGB1 protein (Tilsner et al., 2009), and TGB3 protein also associates with the cortical ER network and MBB (Krishnamurthy et al., 2003; Samuels et al., 2007; Bamunusinghe et al., 2009). Given these findings, the first pathway would indicate that the potexvirus RNP movement complex is released from MBB by TGB3 protein and that the released RNP movement complex then binds to the TGB2/3 granular vesicles in the ER tubule and moves to the PD (Verchot-Lubicz et al., 2010; **Figure 5A**, PATH 1).

The second pathway for the vRNA trafficking of potexvirus to PD by TGB3 protein is supported by interaction and localization assays between TGB2 and TGB3 proteins (**Figure 5A**, PATH 2). BaMV TGB2 protein localizes in the ER structures when expressed in the absence of TGB3 protein, whereas TGB3 protein expressed alone localizes in membrane bodies at the cell periphery (Lee et al., 2010). Bimolecular fluorescence complementation assay has indicated that BaMV TGB2 protein physically interacts with TGB3 protein in a membrane-associated form (Wu et al., 2011). The latter study also reported an interaction between TGB2 protein and either CP or TGB1 protein. The interactions of TGB1–TGB2 and TGB2–CP were associated with the ER network, which is consistent with the localization of TGB2 protein, and these interaction complexes were translocated to the TGB3-containing punctae within ER tubules by co-expression of TGB3 protein (Wu et al., 2011). In another study, which involved the mutation of the C-terminal domain of BaMV CP, the BaMV CP mutant did not interact with the HELD of BaMV replicase, and the mutation severely delayed the virus cell-to-cell movement independent of encapsidation (Lee et al., 2011). Thus, the authors suggested that movement of BaMV from an infected cell to a non-infected cell involves the RNP movement complex containing the replicase, TGB1 protein, CP, and vRNA. In PVX, the replicase is associated with ER membranes and co-localizes with the ER-derived TGB2/3 granular vesicles (Doronin and Hemenway, 1996; Bamunusinghe et al., 2009), indicating that the RNP movement complex containing replicase may be an intermediate form between the VRC and the final RNP movement complex.

Recently, the second model suggesting the stable association of the virion cargo with the TGB2- and TGB3-based membrane complex and recruitment of TGB1 protein to the PD by this complex for cell-to-cell movement of BaMV has been proposed (Chou et al., 2013; **Figure 5B**). The authors suggested that TGB3 protein assists the targeting of the TGB2-induced vesicles or the ER-localized TGB2 protein to the cell periphery (Ju et al., 2005, 2007; Lee et al., 2010) and the movement of virus across cells (Verchot-Lubicz et al., 2010; Wu et al., 2011). TGB3 protein associates with TGB2 protein as a complex and this TGB3-based complex containing TGB2 protein (TGB2/TGB3 complex) stably associates with CP-encapsidated vRNA (virion form) but not partially CP-encapsidated vRNA containing TGB1 protein (non-virion form of RNP movement complex; Chou et al., 2013). They also showed that the stable TGB2–TGB3–virion complex associates with TGB1 protein for targeting to the PD and proposed the refined model for potexvirus vRNA trafficking to PD (Chou et al., 2013). These authors also suggested two pathways in the refined model (**Figure 5B**, PATH 1 and PATH 2) and speculated that TGB2 protein in the membrane-associated TGB2–TGB3–virion complex of BaMV cooperatively interacts with TGB1 protein for efficient PD-localization, while TGB3 protein in the same complex drives the whole movement complex to the cortical ER of cell periphery (Chou et al., 2013).

Interestingly, AltMV TGB3 protein was localized at the chloroplast membrane which may be the main site of AltMV replication and accumulated preferentially in mesophyll cells, whereas PVX TGB3 is localized to the ER and accumulates primarily in the epidermis (Lim et al., 2010a). The AltMV TGB3 interacts with Photosystem II (PSII) oxygen-evolving complex (OEC) protein (PsbO), a nuclear-encoded major component of the chloroplast-localized OEC of PS II, surrounding chloroplast in mesophyll cells. The finding raised the possibility that the interaction possibly draws the chloroplasts together and induces symptom development (Jang et al., 2013). Comparative sequence analyses revealed that AtlMV TGB3 has limited similarity to the TGB3 protein homologs of other potexviruses. Mutational analyses showed that AltMV TGB3 protein contains a novel signal for chloroplast membrane localization. The mutations of the chloroplast-targeting signal in AltMV TGB3 protein resulted in very limited cell-to-cell movement, suggesting that this signal is required for the systemic movement of AltMV (Lim et al., 2010a). Together, these data indicate that the mechanisms for viral movement may differ among potexviruses (Lin et al., 2006).

## INTERCELLULAR TRANSPORT OF POTEXVIRUS vRNA THROUGH THE PD

For transport of vRNA to the adjacent cell, most plant viruses must increase the PD SEL and exit through the PD. As mentioned earlier, potexvirus and plant viruses in general move their vRNA through the PD as its RNP movement complex or virion form. Lough et al. (1998, 2000) showed that TGB1 protein is essential for plasmodesmal gating rather than CP which is involved in RNP movement complex.

In a new model for intercellular transport of PVX vRNA at the entrances of PD at the late stage of infection that was recently proposed by Tilsner et al. (2013), vRNA processing and trafficking are highly compartmentalized at PD, i.e., replication occurs at the PD so that vRNA is rapidly moved through PD and to adjacent cells soon after replication. The authors, who termed the model coreplicational insertion, reported that fluorescent protein–TGB1 and –CP fusions (FP–TGB1 and FP–CP) co-localize in the PD channel with the intercellular wall space, but that FP–TGB2 and FP–TGB3 fusion proteins localize in punctate “caps” at the cytoplasmic orifices of the PD; these punctate caps harbor the replicase and vRNA, such that the CP aligns with the vRNA inside the pores, whereas CP without the vRNA localizes inside the PD (Tilsner et al., 2013). The caps at the PD orifices during PVX infection have been reported to be replication sites containing non-encapsidated vRNA whorls like those observed in VRC (Tilsner et al., 2009, 2013; Tilsner and Oparka, 2012). At 2–3 days post infiltration (dpi), TGB2 protein localizes in the ER tubule or ER-derived granular vesicles (Ju et al., 2005). However, at 1–2 dpi, when expression levels are still low, TGB2 protein is concentrated in punctae along the lateral walls where PD are present rather than in the ER (Tilsner et al., 2013). This finding suggests that TGB2 protein first targets PD and localizes at the ER or ER-associated granules. TGB3 protein also localizes at PD caps with TGB2 protein (Schepetilnikov et al., 2005; Tilsner et al., 2013), and TGB2 protein increases PD-located TGB3 protein (Tilsner et al., 2013). These results support the inference that TGB2 and TGB3 proteins act as a complex (Lee et al., 2010). Thus, Tilsner et al. (2013) have provided convincing evidence that although the three TGB proteins are localized to PD during PVX infection, TGB1 protein is localized inside the pores together with CP while TGB2/3 complexes are localized in caps at the PD orifices (**Figure 3**). In contrast to earlier models, the new model indicates that virus replication and movement are not spatially separated within the cell. However, some questions concerning the interactions between TBG proteins have yet to be experimentally confirmed, i.e., how three TGB proteins cooperate to facilitate vRNA transport (Tilsner et al., 2013) and whether other factors including host protein(s) are required for these interactions and for vRNA transport.

With respect to vRNA transport through the PD at the late stage of infection, the new model indicates that the TGB1 protein at the PD caps first functions as a RNA helicase and diverts progeny vRNA away from replication/translation and toward the assembly of the RNP movement complex (Tilsner et al., 2012, 2013; **Figure 3**). Next, in the presence of locally translated CP, the nascent progeny RNA is encapsidated *in cis*, and TGB1 protein binds specifically to the 5′ terminal CP subunits of partially or fully encapsidated virions (Karpova et al., 2006; Zayakina et al., 2008). Finally, TGB1 protein directs the CP-encapsidated nascent virions as a RNP movement complex into the gated PD pore (**Figure 3**).

## HOST CELLULAR FACTORS INVOLVED IN CELL-TO-CELL MOVEMENT OF POTEXVIRUS

The viral RNP movement complex interacts with host cellular factors at the orifice of the PD, which allows cell-to-cell movement (Morozov and Solovyev, 2003; Verchot-Lubicz, 2005; Lucas, 2006). However, the interactions between the viral RNP movement complex and host cellular factor(s) remain unclear.

In addition to documenting that TGB proteins and CP have crucial roles in potexvirus cell-to-cell movement, recent research has identified and characterized cellular host proteins that might be involved in intercellular movement of potexvirus. The casein kinase 2 family (CK2) phosphorylates PVX TGB1 protein as well as the *Tomato mosaic virus *(ToMV) P30 MP (Matsushita et al., 2003; Modena et al., 2008). Modena et al. (2008) showed that recombinant and native PVX TGB1 protein is phosphorylated by *N. tabacum* extracts from PVX-infected leaves and that PVX TGB1 protein is efficiently phosphorylated by the recombinant tobacco CK2 subunit. Using a phosphopeptide mass mapping approach, they also found that TGB1 protein is phosphorylated at Ser-165 within a CK2 consensus sequence (Modena et al., 2008). Few studies concerning the phosphorylation of plant viral MPs have been reported. Most studies have been performed with the tobamoviruses *Tobacco mosaic virus* (TMV) and ToMV (Citovsky et al., 1993; Kawakami et al., 1999; Waigmann et al., 2000). TMV MP is phosphorylated by a kinase in cell wall-enriched extracts (Waigmann et al., 2000), and the kinase is associated with PD and belongs to the CK1 family (CK1; Lee et al., 2005). Experiments with phosphorylation mutants have shown that phosphorylation of TMV MP negatively regulates viral transport through PD in a host-dependent manner (Waigmann et al., 2000). A recent study showed that tobacco Serine/threonine kinase-like protein NbSTKL, which is a membrane-associated protein, is involved in the cell-to-cell movement of the potexvirus BaMV (Cheng et al., 2013); that finding suggests the possibility that membrane-associated host proteins interact with the TGB1-containing RNP movement complex and aid cell-to-cell trafficking of potexviruses. However, how host kinase proteins directly or indirectly affect the assembly of the RNP movement complex and/or PD gating has not been studied.

A yeast two-hybrid screening assay has also identified three host proteins (TIP1, TIP2, and TIP3) that interact with PVX TGB2 protein (Fridborg et al., 2003). These proteins interact with β-1,3-glucanase to regulate the degradation of PD-accumulated callose and to increase the PD SEL (Fridborg et al., 2003). PVX TGB1 protein interacts with remorin (REM), a protein located in the cytosolic leaflet of plasma membrane microdomains (lipid rafts) and in clusters at PD. REM can be phosphorylated in the presence of oligogalacturonides and can serve as a counteracting membrane platform for docking of the viral RNP movement complex (Raffaele et al., 2009). In addition, when REM was overexpressed in plants, it inhibited the movement of PVX through PD. This finding suggests that membrane rafts might have a critical role in virus intercellular trafficking.

A recent study suggested that several host cellular heat shock proteins (Hsps), which function as chaperones and folding enzymes, are also related to potexvirus movement (Verchot, 2012). Hsps are conserved molecular polypeptide chaperones that primarily ensure protein quality. Potexviruses rely on chaperone systems in the ER to support cell-to-cell movement. TGB3 protein induces the expression of ER-resident chaperones via the bZIP60 transcription factor (Verchot, 2012). The CP of another potexvirus, *Pepino mosaic virus*, interacts with Hsp cognate 70 (Hsc70), which has APTase activity, and it seems to take the translocation ability of CP-Hsc70 interaction to facilitate viral cell-to-cell movement through the PD (Mathioudakis et al., 2012). In addition, another host cellular protein, named NbPCIP1, was characterized from a *Nicotiana benthamiana *cDNA library as a PVX CP-interacting protein based on the yeast two-hybrid system. NbPCIP1 enhanced PVX replication and movement in *N. benthamiana* (Park et al., 2009).

Overall, it is quite clear that the identified host cellular proteins play important roles in potexvirus replication and cell-to-cell movement. However, many of them still remain unclear and need to be elucidated in regard to identifying and functionally characterizing host cellular proteins associated with the infection cycles of potexviruses.

## HOST CELLULAR FACTORS INTERACTING WITH VIRAL RNA ELEMENTS IN POTEXVIRUS MOVEMENT

The (+) SL1 RNA or (-) SL1 RNA of PVX behaves as a *cis*-acting element and can affect cell-to-cell movement of PVX (Lough et al., 2006). As noted earlier, many host proteins that interact with viral proteins and that might affect intra-/inter-cellular trafficking of potexviruses have been identified. Little is known, however, about how host proteins interact with vRNAs because functional studies of plant host proteins usually concern interactions between viral protein and host protein rather than between vRNA and host protein. In this regard, PVX SL1 RNAs can efficiently bind to proteins extracted from tobacco protoplasts, as confirmed by a systematic evolution of ligands by exponential enrichment *in vitro* (Kwon and Kim, 2006). Northwestern blot analysis and matrix-assisted laser desorption ionization time-of-flight mass spectrometry have been recently used to identify tobacco proteins that bind to PVX SL1 RNAs (Cho et al., 2012a).

The host protein NbDnaJ (or Hsp40) binds to SL1 (-) RNA of PVX and also interacts with PVX CP. As indicated by deletion assay, the C-terminal region of NbDnaJ is essential for the interaction with PVX CP (Cho et al., 2012c). According to the latter study, when NbDnaJ is overexpressed in transient assay, PVX movement is suppressed. This study showed that NbDnaJ plays a role in the early stages of viral infection by suppressing PVX replication and movement. A previous study had identified another host protein, NbMPB2Cb, that binds with both SL1 (-) and SL1 (+) RNAs of PVX (Cho et al., 2012b). NbMPB2Cb localizes at microtubules in the ER and suppresses PVX movement (Cho et al., 2012b). Although it is not immediately relevant to the intra-/intercellular movement of potexviruses, SL1 (+) RNA binds to the *N. tabacum *WRKY1 transcription factor (NtWRKY1; Kim et al., 2002; Park and Kim, 2012). By increasing PVX accumulation, silencing of NtWRKY1 in *N. benthamiana *caused plants to exhibit lethal apical necrosis, suggesting that NtWRKY1 regulates multiple defense response genes (Park and Kim, 2012). Together, these results suggest that a plant vRNA element greatly affect viral movement and replication, possibly by interacting with several host factors. The latter study might be the first to report that the interaction between a plant vRNA element and a host cellular protein is required for viral movement.

## CONCLUDING REMARKS AND FUTURE PERSPECTIVES

Substantial information has been obtained concerning the cellular mechanisms underlying intra- and intercellular movement of plant RNA viruses. Here, we have summarized the intracellular and intercellular movement of vRNA in potexviruses based on recent studies. As indicated, intracellular and intercellular vRNA movement is mediated by viral MPs and host proteins, indicating that the plasmodesmal transport of vRNA is a highly regulated process. Here, we have tried to summarize the cell-to-cell movement of potexvirus vRNA with intracellular trafficking and intercellular transport of vRNA in plant cells. Although we have provided two general models that describe how potexvirus vRNA is trafficked to PD (**Figure 5**; Verchot-Lubicz et al., 2010; Chou et al., 2013), TGB protein interactions and many other aspects of intra- and intercellular potexvirus movement remain unclear. To further investigate the interactions between TGBs in relation to potexvirus movement, additional experimental evidence clarifying whether each TGB directly binds to the other TGBs or requires other factors at each step during the movement process(es) is necessary. As for the possible involvement of the SL1 of PVX and other *cis*-acting elements for potexvirus movement, we should determine whether viral and host cellular proteins compete for binding to the viral element and how viral elements regulate the viral movement.

The viral RNP movement complex interacts with host cellular factors at the orifice of the PD, which enables cell-to-cell movement (Morozov and Solovyev, 2003; Verchot-Lubicz, 2005; Lucas, 2006). Although it is well-known that host proteins interacting with PVX TGB2 protein (TIP1, TIP2, and TIP3) interact with β-1,3-glucanase to regulate the degradation of PD callose and to increase the PD SEL (Fridborg et al., 2003), the interactions between the viral RNP movement complex and host cellular factor(s) remain unclear. Little is also known about how potexviruses interact with host cellular proteins to mediate virus movement. Identifying new host cellular proteins required for cell-to-cell movement should be facilitated by the use of microarrays and/or RNA-Seq with high-throughput gene silencing and other new methods.

It also appears that potexviruses do not always have the same movement mechanisms even if all have TGB proteins (Lim et al., 2010a). Thus, research is needed on different viruses in the same genus. Research is also needed regarding the functions of PD that mediate vRNA movement and how PD-associated proteins regulate vRNA movement through PD. In addition to increasing our understanding of the virus infection cycle, understanding the details of virus movement is valuable because it provides insight into fundamental aspects of the cellular machinery. We suspect that potexviruses will continue to be useful models for studying intra- and inter-cellular vRNA movement in plants.

## Conflict of Interest Statement

The authors declare that the research was conducted in the absence of any commercial or financial relationships that could be construed as a potential conflict of interest.
